# Maternal and Neonatal Outcomes and Health System Costs in Standard Public Maternity Care Compared to Private Obstetric‐Led Care: A Population‐Level Matched Cohort Study

**DOI:** 10.1111/1471-0528.18286

**Published:** 2025-07-14

**Authors:** Emily J. Callander, Joanne Enticott, Ben W. Mol, Shakila Thangaratinam, Jenny Gamble, Stephen Robson, Helena Teede

**Affiliations:** ^1^ Monash Centre for Health Research and Implementation Monash University Melbourne Australia; ^2^ School of Public Health University of Technology Sydney Sydney Australia; ^3^ Department of Obstetrics & Gynaecology, School of Clinical Sciences Monash University Melbourne Australia; ^4^ Institute of Life Course and Medical Sciences University of Liverpool Liverpool UK; ^5^ Liverpool Women's National Health Service Foundation Trust Liverpool UK; ^6^ Centre for Healthcare and Communities Coventry University Coventry UK; ^7^ School of Nursing and Midwifery Monash University Melbourne Australia; ^8^ College of Health and Medicine Australia National University Canberra Australia

**Keywords:** maternity, models of care, value

## Abstract

**Objective:**

We aimed to compare health outcomes and costs in standard public maternity care compared to private obstetric‐led maternity care.

**Design:**

Observational study with linked administrative data.

**Setting:**

Australian maternity care.

**Population:**

867 334 births, covering all births in three states of Australia between 2016 and 2019.

**Methods:**

Standard public care involved mainly fragmented midwifery, obstetric and General Practitioner provider care, with birth in a public hospital. Private obstetric‐led care was led by a personally selected obstetrician, with midwifery involvement and birth in a private hospital. We analysed outcomes from pregnancy onset to 4 weeks post‐birth. Matching was utilised to account for demographic, socio‐economic and clinical characteristics.

**Main Outcome Measures:**

Stillbirths or neonatal deaths; neonatal intensive care admissions; APGAR score < 7 at 5 min; 3rd or 4th degree perineal tears; maternal haemorrhages; mean cost per pregnancy episode.

**Results:**

Higher adverse outcomes in standard public maternity care compared to private obstetric‐led care, including 778 more stillbirths or neonatal deaths (OR 2.0, 95% CI: 1.8–2.1), 2747 more APGAR score < 7 at 5 min (OR 2.0, 95% CI: 2.0–2.1), 3273 more 3rd or 4th degree perineal tears (OR 2.9, 95% CI: 2.7–3.1) and 10 627 additional maternal haemorrhages (OR 2.7, 95% CI: 2.6–2.8). Mode of birth correlated with neonatal death. Mean cost to all funders in Australian dollars per pregnancy episode was $5929 higher in standard public maternity care.

**Conclusion:**

We have shown significantly lower adverse health outcomes and costs in private obstetric‐led care compared to standard public maternity care.

## 1 Introduction

There has been a rapid rise in interrelated, interdependent vulnerabilities and inequitably driving adverse pregnancy outcomes [1]. These include escalating obesity, which aligns with broader societal trends, driven by eco‐social factors and concentrated in disadvantaged populations [1]. These factors adversely impact maternal and neonatal outcomes, impacting families and increasing health system and economic burden, highlighting the need for adaptive, responsive value‐based healthcare across the domains of patient and provider experience, quality of care and outcomes, efficiency and sustainability, considering vulnerabilities and founded on the tenet of universal healthcare for all [2].

Flawed approaches to maternity care, such as the United Kingdom Shrewsbury National Health Service (NHS) Trust, East Kent NHS and Australian Mackay hospital service failures [3, 4, 5], have delivered poor pregnancy outcomes, with independent inquiries highlighting failure to investigate, learn and improve. Improvements include the midwifery continuity model of care, which compared to standard maternity care in public healthcare settings [6], improve outcomes and cost‐effectiveness, yet scale‐up remains challenging. There is otherwise a dearth of exploration of broader models of care. In this context, operationalising existing administrative data can enable other models of care across population, provider, continuity and setting to be explored, with outcomes and costs identified. This is key to the investigation, learning and improvement of maternity care [7].

Operationalising data in a health system that learns and improves is especially relevant to complex maternity models of care in Australia, which includes universal public healthcare supplemented by private care, with embedded mandatory maternity data reporting. Leveraging our unique linked population‐level administrative datasets, here we aimed to (i) quantify differences in value‐based care across health outcomes and costs between standard public maternity care and private obstetric‐led care in Australia, comparing cohorts matched for demographic, socio‐economic and clinical characteristics and (ii) explore relationships between potential drivers and adverse outcomes. Women giving birth in standard public maternity care (‘standard maternity care’), receive highly variable care across providers, settings and populations including clinical risks, with mainly fragmented provider care involving midwives, obstetricians and General Practitioners, and birth in a public hospital. ‘Private obstetric‐led care’ is led by a personally selected, named obstetrician, with midwifery involvement and birth in a private hospital.

## 2 Methods

### 2.1 Study Design and Participants

This retrospective observational study was conducted in the Australian healthcare system, which aims for equity of access as a fundamental principle, delivered through a dual public and private care system [8]. Of the ~300,000 births annually, around 75% are in standard care with births in public hospitals. In terms of providers, antenatal, intrapartum and postnatal care is largely fragmented including public hospital midwives, obstetricians, general practitioners (‘Shared care’) and trainees. A less common provider model is midwifery group practice caseload antenatal, intrapartum and postnatal care by a named public hospital midwife in collaboration with public hospital doctors [9, 10]. The private obstetric‐led care model serves around 24% of Australian births, with birth in private hospitals. Antenatal, intrapartum and postnatal care is provided by a personally selected obstetrician [10], with midwives variably involved in care. Birth at home, births with private midwives in private hospitals and births in private hospitals with public funding were excluded. The funding of health services in Australia and relationship to providers is described in Appendix S1.

### 2.2 Data Source

The analysis comparing outcomes used the unique Maternity2000 linked dataset, linking routine administrative data on births (covering the pregnancy, birth and post‐partum) occurring between January 2016 and December 2019, across the three most populous states in Australia including 78% of annual births nationally. Women giving birth and their babies were identified from the Perinatal Data Collection, a mandatory dataset of all births, which includes women's demographic and clinical characteristics prior to and during pregnancy, birth and post‐partum. These data were linked to multiple inpatient, outpatient and emergency department datasets across all private and public hospitals for both the woman and neonate, from onset of pregnancy to 4 weeks post‐partum.

The analysis comparing costs used the unique Maternity1000 dataset [11]. This linked data was for all births occurring between January 2016 and June 2018 in one Australian state with 21% of annual births nationally. Women giving birth and their babies were identified from the Perinatal Data Collection, with linkage to the same datasets as Maternity2000, again from onset of pregnancy to 4 weeks post‐partum. These data were additionally linked to the Medicare and Pharmaceutical Benefits Scheme (PBS) claims records, covering all non‐hospital services and prescription pharmaceutical use.

### 2.3 Birth Outcomes

Foetal and neonatal outcomes included: stillbirth; neonatal death (death within 28 days of birth); admission to neonatal intensive care unit (NICU); APGAR score < 7 at 5 min; APGAR score < 4 at 5 min; birthweight in 5th centile or less; birthweight in 6th–10th centile; hypoxic–ischaemic encephalopathy (ICD‐10‐AM code block: P91); birth trauma—including brachial plexus injury, fractured clavicle or humerus or other long bones, peripheral nervous system damage (ICD‐10‐AM code block: P13.3, P13.4, P14); intrauterine hypoxia (ICD‐10‐AM code block: P20); other perinatal morbidity—meconium aspiration syndrome, congenital pneumonia or respiratory distress syndrome (ICD‐10‐AM code block: P24.0, P22, P23). Maternal birth outcomes included: perineal damage—3rd/4th degree tear; haemorrhage in the woman (ICD‐10‐AM code block: O67, O72); retained placenta (ICD‐10‐AM code block: O73); and rupture of uterus (ICD‐10‐AM code block: O71). NICU admission was unavailable for one state.

### 2.4 Identification of Costs

All health service use covering pregnancy, birth and to 4 weeks post‐partum was identified from Maternity1000. Costs for inpatient, outpatient and emergency department hospital use were based upon the activity‐based funding code assigned for hospital funding purposes to each episode of care, and the corresponding cost per episode reported in the National Hospital Cost Data Collection for public episodes of care and the Private Hospital Bureau Annual Reports for private episodes of care [12, 13]. Costs for Medicare services and prescription medications covered by the PBS were identified directly from the Medicare and PBS claims records, which record the actual amount paid by government and patients through out‐of‐pocket fees for every service and pharmaceutical. Costs were summed and presented by different funders: Public hospital funders (Federal and state governments); Medicare (Federal government); PBS (Federal government); Private health insurers; and Individuals through out‐of‐pocket fees [11]. Costs were adjusted for inflation and presented in 2021/22 Australian dollars (AUD) ($1 = 0.53 Great British Pounds and 0.67 United States Dollars).

### 2.5 Matching Populations

Matching was used to control for key baseline differences between the populations of women who gave birth in standard maternity care and private obstetric‐led care, in both datasets. Matching aims to simulate randomisation by balancing observed baseline covariates between groups in cohort studies [14, 15, 16, 17]. Matching was selected over propensity score matching (PSM) and regression, as it is less sensitive to mis‐specification and extreme values compared to PSM [18] and unlike regression analyses, it does not require assumptions about relationships between confounders and outcomes. Hence, matching is the preferred method when many confounders require adjustment [19]. To demonstrate robustness, a sensitivity analysis was conducted using logistic regression on the full dataset, adjusting for the matching variables.

Matching using simple random sampling, without replacement from women who gave birth in standard maternity care, was conducted based on the number of women who gave birth in private obstetric‐led care in each stratum group of age, body mass index (BMI), born in a non‐English speaking country, socio‐economic status, rurality of residence, identification as Aboriginal or Torres Strait Islander, smoking after 20 weeks' gestation, parity, plurality, Assisted Reproductive Technology (ART) use, and pre‐existing diabetes, gestational diabetes, hypertension or preeclampsia. ART and BMI were not available for matching for one state, and smoking for another. Analysis was conducted on the complete dataset, with those with missing data (*n* = 116) excluded. This produced two equal‐sized samples with the same distribution of characteristics in the stratum.

### 2.6 Statistical Analysis

Descriptive statistics were completed for demographic and clinical characteristics using the matched cohort from Maternity2000.

Odds ratios of the likelihood of each outcome were calculated on matched cohorts. Bootstrapping with 50 rematched datasets was conducted, and the odds ratios produced with each dataset assessed. This bootstrapping generated 50 samples with different matched pairings, ensuring that the findings are not spurious and do not arise from a single matched sample.

Multiple sub‐group analyses were run with the data limited to (1) an intention to treat population, where births at > 28 weeks, with women who gave birth in a public hospital with no private obstetric consultations, were compared to women who initiated care with a private obstetrician prior to 20 weeks' gestation (any women who were transferred to care in the public system were still classified as being in the private obstetric‐led model of care); and (2) a very low‐risk population including only women between 21 and 35 years, healthy BMI (18.50–24.99 kg/m^2^), non‐smoker after 20 weeks' gestation, singleton pregnancy, no ART use, and no pre‐existing diabetes, gestational diabetes, hypertension or preeclampsia, stratified by nulliparous and multiparous women. The matching was then reconducted and results generated. Further sensitivity analysis stratified outcomes by (1) nulliparous women; (2) multiparous women; (3) nulliparous women giving birth at ≥ 37 weeks' gestation; (4) multiparous women giving birth at ≥ 37 weeks' gestation; (5) infants born at different gestational ages; and (6) infants born at different gestational ages with and without congenital abnormalities. Results were also stratified by area‐based socio‐economic quintile. The role of earlier birth and caesarean section delivery in mitigating the relationship between private obstetric‐led care and stillbirth or neonatal death was explored in a stepped logistic regression model, adding preterm birth and caesarean section to the model iteratively.

Differences in mean cost between standard maternity care and private obstetric‐led care in the matched cohorts were analysed. Bootstrapping with 50 rematched datasets was conducted, and the means and difference in means between the two groups produced with each dataset assessed. Three sub‐group analyses were run on the analysis of costs, to assess the difference in (1) women who gave birth in a public hospital with no private obstetric consultations compared to women who initiated care with a private obstetrician prior to 20 weeks' gestation and gave birth in either a public or private hospital; (2) in a very low‐risk population (defined above); and (3) with NICU admissions excluded, given its high cost. Matching was then repeated. Results were stratified by area‐based socio‐economic quintile.

Descriptive statistics of the number of stillbirths and neonatal deaths that occurred in different birthweight centile groups, gestational age groups, with congenital anomalies and with an APGAR score of < 4 at 5 min. An analysis of the likelihood of neonatal death was conducted with multivariable logistic regression, using mode of birth, onset of labour, private obstetric‐led care, age group, BMI group, born in a non‐English speaking country, geographically based socio‐economic status, rurality of residence, identification as Aboriginal or Torres Strait Islander, smoking after 20 weeks' gestation, parity, plurality, ART use, pre‐existing diabetes or gestational diabetes, hypertension and preeclampsia as covariates. This analysis was completed on the full matched cohort, across both models of care. SAS 9.4 was used for all analysis.

## 3 Results

### 3.1 Outcomes Analysis

Overall, there were 661 455 births in standard maternity care and 202 236 births in private obstetric‐led care in the 4‐year study period in the three States. In the full sample, before matching, women giving birth in standard maternity care were younger, had a higher BMI, were more likely to identify as Aboriginal or Torres Strait Islander, be born in a non‐English speaking country, smoke after 20 weeks' gestation and be of lower socio‐economic status (Table 1). Matching produced two equal cohorts of 184 146 women in standard and private obstetric‐led care, with similar demographic, socio‐economic and clinical characteristics (Table 1).

**TABLE 1:** Demographic characteristics of women before and after matching, all women giving birth in three states, 2016–2019.

	Pre‐matching				Post‐matching			
	Standard maternity care (*n* = 661 455)		Private obstetric‐led care (*n* = 202 236)		Standard maternity care (*n* = 184 146)		Private obstetric‐led care (*n* = 184 146)	
	*N*	%	*N*	%	*N*	%	*N*	%
Age group								
< 20 years	23 692	3.6%	289	0.1%	225	0.1%	225	0.1%
21–35 years	481 464	73.0%	122 018	59.8%	114 741	62.3%	114 741	62.3%
> 35 years	154 511	23.4%	81 717	40.1%	69 174	37.6%	69 174	37.6%
Mean age (SD)	30.4 (5.5)		33.6 (4.3)		32.3 (5.2)		33.4 (4.2)	
BMI group^a^								
Underweight	16 417	4.6%	6489	5.4%	4835	4.8%	4835	4.8%
Healthy range	168 666	46.8%	67 867	56.8%	58 233	57.5%	58 233	57.5%
Overweight	90 942	25.3%	27 444	23.0%	23 420	23.1%	23 420	23.1%
Obese	84 136	23.4%	17 601	14.7%	14 733	14.6%	14 733	14.6%
Mean BMI (SD)	34.8 (89.5)		26.6 (41.5)		30.2 (70.3)		26.7 (41.9)	
ATSI	36 648	5.6%	1002	0.5%	807	0.4%	807	0.4%
Born in non‐English speaking country	200 723	30.4%	53 534	26.2%	50 361	27.4%	50 361	27.4%
Smoker	59 784	9.1%	956	0.5%	717	0.4%	717	0.4%
Nulliparous	253 231	38.4%	89 423	43.8%	81 487	44.3%	81 487	44.3%
Singleton pregnancy	641 123	97.2%	197 552	96.8%	179 369	97.4%	179 369	97.4%
ART utilised^a^	9683	2.7%	14 810	12.4%	7194	7.1%	7194	7.1%
Socio‐economic quintile (most to least disadvantaged)								
1	146 981	22.3%	14 263	7%	13 498	7.3%	13 498	7.3%
2	146 067	22.2%	22 432	11%	20 281	11.0%	20 281	11.0%
3	146 940	22.2%	40 060	19.7%	37 986	20.6%	37 986	20.6%
4	127 159	19.3%	51 308	25.2%	47 801	26.0%	47 801	26.0%
5	88 040	13.4%	74 117	36.3%	63 686	34.6%	63 686	34.6%
Rurality of residence								
Major city	464 029	70.3%	173 613	85.1%	158 688	86.2%	158 688	86.2%
Inner regional	123 944	18.8%	18 260	9.0%	15 292	8.3%	15 292	8.3%
Outer regional	52 236	7.9%	7422	3.6%	6584	3.6%	6584	3.6%
Rural	7344	1.1%	953	0.5%	807	0.4%	807	0.4%
Diabetes	90 033	13.7%	20 337	10.0%	18 370	10.0%	18 370	10.0%
Hypertension	18 417	2.8%	6371	3.1%	5272	2.9%	5272	2.9%
Preeclampsia^a^	7553	2.1%	2022	1.7%	1460	1.4%	1460	1.4%

Abbreviations: ART, Assisted Reproductive Technology; ATSI, Aboriginal or Torres Strait Islander; BMI, body mass index; SD, standard deviation.

^a^ Not available for one state.

In standard maternity care, there were 778 more stillbirths or neonatal deaths (0.9% versus 0.4%; OR 2.0 [95% CI: 1.8–2.1]), 2301 more babies admitted to NICU (3.5% vs. 1.3%; OR 2.9 [95% CI: 2.7–3.0]), 2747 more babies with APGAR score < 7 at 5 min (3.0% vs. 1.5%; OR 2.0 [95% CI: 2.0–2.1]) compared to private obstetric‐led care (Table 2). In standard maternity care, there were also 3273 more women with 3rd or 4th degree perineal tears (2.5% vs. 0.7%; OR 2.9 [95% CI: 2.7–3.1]), and 10 627 more with haemorrhage (9.6% vs. 3.8%; OR 2.7 [95% CI: 2.6–2.8]) compared to private obstetric‐led care. All clinical outcomes show an effect favouring private obstetric‐led care versus standard maternity care (Table 2), with a binomial probability of this occurring by chance of 0.0001 (1 in 8192). Mode of birth is presented in Table 2, showing lower caesarean sections (31.6% vs. 47.9%, OR 0.5 [95% CI: 0.5–0.5]) and vaginal birth with vacuum (7.2% vs. 9.9%, OR 0.5 [95% CI: 0.5–0.5]), but higher induction of labour (32.5% vs. 31.1%, OR 1.1 [95% CI: 1.1–1.1]) in standard maternity care. Birth between 34 and 37 weeks was lower in standard maternity care (5% vs. 6.1%, OR 0.8 [95% CI: 0.8–0.8]); as was birth between 37 and 39 weeks (24.8% vs. 39.8%, OR 0.5 [95% CI: 0.5–0.5]).

**TABLE 2:** Clinical birth outcomes of women and babies, matched cohort of women giving birth in standard maternity care and private obstetric‐led care in three states 2016–2019.

Clinical outcomes	Matched results						Bootstrapped results^d^	Intention to treat analysis^e^
	Standard maternity care (*n* = 188 493)		Private obstetric‐led care (*n* = 188 493)		Excess in standard maternity care	OR (95% CI)	OR (95% CI)	OR (95% CI)
	*N*	%	*N*	%				
Stillbirth or neonatal death***	1584	0.9%	806	0.4%	778	2.0 (1.8–2.1)	2.0 (1.9–2.1)	4.5 (2.7–7.6)
Stillbirth***	1103	0.6%	634	0.3%	469	1.7 (1.6–1.9)	1.8 (1.7–1.8)	Not reportable
Neonatal death***	481	0.3%	172	0.1%	309	2.8 (2.4–3.3)	2.8 (2.6–3.0)	Not reportable
Neonatal intensive care admission^a^ ^,^ ***	3584	3.5%	1283	1.3%	2301	2.9 (2.7–3.0)	2.9 (2.8–2.9)	4.4 (3.7–5.1)
APGAR score < 7 at 5 min***	5448	3.0%	2701	1.5%	2747	2.0 (2.0–2.1)	2.0 (2.0–2.0)	1.9 (1.7–2.2)
APGAR score < 4 at 5 min***	934	0.9%	565	0.6%	369	1.7 (1.5–1.8)	1.7 (1.6–1.8)	3.2 (2.2–4.6)
Birthweight, < 5th centile***	9224	5.0%	6806	3.7%	2418	1.4 (1.3–1.4)	1.4 (1.2–1.4)	1.3 (1.2–1.4)
Birthweight, 5th–10th centile***	9203	5.0%	8186	4.5%	1017	1.1 (1.1–1.2)	1.1 (1.1–1.2)	1.1 (1.0–1.2)
Hypoxic ischaemic encephalopathy***	103	0.1%	49	0.0%	54	2.1 (1.5–3.0)	1.9 (1.6–2.1)	Not reportable
Trauma***	140	0.1%	46	0.0%	94	3.0 (2.2–4.2)	2.8 (2.4–3.2)	4.9 (4.1–6.0)
Hypoxia***	560	0.3%	110	0.1%	450	5.1 (4.2–6.3)	5.3 (5.0–5.5)	7.0 (3.8–12.8)
Other***	10 486	5.7%	6372	3.5%	4114	1.7 (1.6–1.7)	1.7 (1.7–1.7)	1.4 (1.3–1.5)
Perineal damage^b^ ^,^ ***	4557	2.5%	1284	0.7%	3273	2.9 (2.7–3.1)	2.9 (2.9–3.0)	2.9 (2.5–3.3)
3rd degree tear***	4324	2.4%	1205	0.7%	3119	2.9 (2.7–3.1)	2.9 (2.9–3.0)	Not reportable
4th degree tear***	233	0.1%	79	0.0%	154	2.3 (1.8–3.0)	2.3 (2.1–2.5)	Not reportable
Maternal haemorrhage***	17 594	9.6%	6967	3.8%	10 627	2.7 (2.6–2.8)	2.7 (2.6–2.7)	1.9 (1.8–2.0)
Ruptured uterus*	84	0.1%	50	0.0%	34	1.7 (1.2–2.4)	1.9 (1.7–2.2)	Not reportable
Retained placenta***	2198	1.2%	615	0.3	1583	3.6 (3.3–3.9)	3.6 (3.5–3.7)	1.0 (0.8–1.3)
Birth interventions								
Induction of labour***	59 853	32.5%	57 289	31.1%	2564	1.1 (1.1–1.1)	1.1 (1.1–1.1)	
Caesarean section—any***	58 220	31.6%	88 139	47.9%	−29 919	0.5 (0.5–0.5)	0.5 (0.5–0.5)	
Planned caesarean section***	34 203	18.6%	65 232	35.4%	−31 029	0.4 (0.4–0.4)	0.4 (0.4–0.4)	
Unplanned caesarean section^c^ ^,^ ***	24 023	16.0%	22 909	19.3%	1114	0.8 (0.8–0.8)	0.8 (0.8–0.8)	
Vaginal birth with forceps***	12 166	6.6%	8322	4.5%	3844	1.1 (1.1–1.2)	1.5 (1.5–1.5)	
Vaginal birth with vacuum***	13 188	7.2%	18 228	9.9%	−5040	0.5 (0.5–0.5)	0.7 (0.7–0.7)	
Preterm birth								
Gestational age at birth < 34 weeks	4818	2.6%	3007	1.6%	1811	1.6 (1.5–1.7)	1.6 (1.6–1.6)	
Gestational age at birth 34–37 weeks	9183	5.0%	11 278	6.1%	−2095	0.8 (0.8–0.8)	0.8 (0.8–0.8)	
Gestational age at birth 37–39 weeks	45 747	24.8%	73 290	39.8%	−27 543	0.5 (0.5–0.5)	0.5 (0.5–0.5)	
Gestational age at birth > 39 weeks	124 398	67.6%	96 571	52.4%	27 827	1.9 (1.9–1.9)	1.9 (1.9–1.9)	
Gestational age at birth, mean (SD)	38.9 (2.2)		38.6 (1.7)					

Abbreviations: CI, confidence interval; OR, odds ratio; SD, standard deviation.

^a^ Neonatal intensive care admission data for one state not available.

^b^ Perineum damage denominator excludes those who had a planned caesarean section.

^c^ Caesarean section—unplanned denominator excludes births with planned caesarean section.

^d^ Bootstrapping generated 50 samples with variations in matched pairings, ensuring that the findings are not spurious and do not arise from a single matched sample.

^e^ Based on model of care at 28 weeks, one state, only births > 28 weeks.

* Significant at the < 0.05 level.

*** Significant at the < 0.0001 level.

Bootstrapping with 50 rematched samples yielded similar results (Table 2). Sub‐group analysis on intention to treat in private obstetric‐led care was completed in one state with available data. Here, women who initiated private obstetric‐led care and then transferred to public care were classified in the private obstetric‐led model of care, and results were similar to the primary analysis (Table 2). Further sub‐group analysis showed consistent findings across maternal risk and parity, and for infants born over 28 weeks and at 37 weeks gestation and above, without congenital abnormalities (Tables S1 and S2). Stratifying by socio‐economic quintile, the odds of poor neonatal and maternal outcomes remained consistently higher in standard maternity care compared to private obstetric‐led care (Table S3). Sensitivity analysis using multivariable logistic regression instead of matching produced similar findings (Table S4).

In a stepped logistic regression model of the odds ratio of stillbirth or neonatal death for standard maternity care compared to private obstetric‐led care, adding preterm birth and caesarean section birth to the model did not meaningfully change the odds ratio (Table S5).

### 3.2 Cost Analysis

Overall, there were 110 041 births in standard maternity care and 38 425 births in private obstetric‐led care, in the one state with linked available cost data (Table S6). Matching produced two equal‐size cohorts of 33 857 women with similar characteristics (Table S6).

**FIGURE 1:**
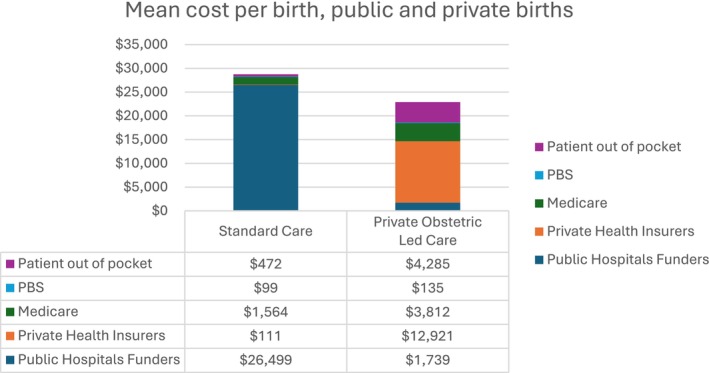
Mean cost per birth to different funders, matched cohort of women giving birth in standard maternity care and private obstetric‐led care in one state, 2016–2018. PBS, Pharmaceutical Benefits Scheme.

Mean costs for each pregnancy, birth and post‐partum episode in AUD were $28 645 (95% CI: $28 417–28 874) for standard maternity care and $22 757 (95% CI: $22 624–$22 890) for private obstetric‐led care (Table 3). These total costs are the cost to all funders of care. For standard maternity care, this total constitutes $26 499 to public hospital funders, $111 to private health insurers, $1564 to Medicare, $99 to Phamaceutical Benefits Scheme and $472 in patient out‐of‐pocket fees (Table 3). For private obstetric‐led care, the total constitutes $1739 to public hospital funders, $12 921 to private health insurers, $3812 to Medicare, $135 to the Pharmaceutical Benefits Scheme and $4285 in patient out‐of‐pocket fees. Bootstrapped analysis demonstrated that mean costs to all funders per pregnancy, birth and post‐partum episode was $5929 (95% CI: $5789–$6081) higher for standard maternity care compared to private obstetric‐led care (Table 3). Figure 1 shows the difference in average cost to different funders, reflecting the different funding pools for standard maternity care versus private obstetric‐led care. Based upon the annual number of births in Australia (315 507), if 25% of women currently accessing private obstetric‐led care were to move to standard maternity care, net costs to government funders (public hospital funders and Medicare) would be $1.77 billion higher per year.

**TABLE 3:** Mean cost per birth to different funders, matched cohort of women giving birth in standard maternity care and private obstetric‐led care in one state, 2016–2018.

Costs	Matched results		Bootstrapped results^a^
	Standard maternity care (*n* = 33 857)	Private obstetric‐led care (*n* = 33 857)	Difference (95% CI)
	Mean (95% CI)	Mean (95% CI)	
Total cost	$28 645 (28 417 to 28 847)	$22 757 (22 624 to 22 890)	$5929 (5789 to 6081)
Public hospitals funders	$26 499 (26 273 to 26 724)	$1739 (1629 to 1849)	$24 867 (24 724 to 25 015)
Private health insurers	$111 (99 to 122)	$12 921 (12 862 to 12 981)	−$12 827 (−12 838 to −12 816)
Medicare	$1564 (1552 to 1576)	$3812 (3791 to 3832)	−$2261 (−2268 to −2255)
Pharmaceutical Benefits Scheme	$99 (92 to 107)	$135 (128 to 143)	−$36 (−42 to −28)
Patient out‐of‐pocket	$472 (463 to 481)	$4285 (4266 to 4304)	−$3850 (−3856 to −3845)

Abbreviation: CI, confidence interval.

^a^ Bootstrapping generated 50 samples with variations in matched pairings, ensuring that the findings are not spurious and do not arise from a single matched sample.

In the sub‐group where women who gave birth in a public hospital with no private obstetric consultations were compared to women who initiated care with a private obstetrician prior to 20 weeks' gestation, mean costs per birth remained comparatively higher for standard maternity care ($28 391, 95% CI: $20 104–$31 448 vs. $23 432, 95% CI: $18 628–$25 479). In the sub‐group analysis limited to very low‐risk women, mean costs per birth remained comparatively higher for standard maternity care for both nulliparous women and multiparous women (Table S7). In sensitivity analysis excluding births with a NICU admission, costs remained higher in standard maternity care (Table S7). Costs also remained consistently higher when stratified by socio‐economic status for women in standard maternity care than private obstetric‐led care (Table S8).

### 3.3 Associations With Adverse Outcomes

In the overall matched cohort of 376 986 births across three states, factors associated with stillbirth or neonatal death are identified in Table 4. Vulnerabilities including maternal BMI category, age and maternal birth in a non‐English speaking country were associated with birth outcomes. Mode of birth was associated with stillbirth or neonatal death, with caesarean section having an adjusted odds ratio (aOR) of 0.3 (0.3–0.4) and induction having an aOR of 2.5 (2.2–2.8). Private obstetric‐led care had an aOR of 0.7 (0.7–0.8) for stillbirth or neonatal death. Higher stillbirths and neonatal deaths in standard maternity care than in private obstetric‐led care were observed across all birthweight centiles and gestational age groups, those with congenital anomalies and those with an APGAR score of < 4 at 5 min (Table S9).

**TABLE 4:** Adjusted odds ratio of stillbirth or neonatal death in matched cohorts of women giving birth in three states 2016–2019.

	OR (95% CI)
Private obstetric‐led care	0.7 (0.7–0.8)
Caesarean section birth	0.3 (0.3–0.4)
Induction of labour	2.5 (2.2–2.8)
Age group	
Less than 20 years	2.8 (1.4–5.7)
21–35 years	Reference
More than 35 years	1.3 (1.2–1.5)
BMI group	
Underweight	1.6 (1.3–2.0)
Healthy range	Reference
Overweight	1.1 (1.0–1.3)
Obese	2.1 (1.9–2.4)
Mother identifies as Aboriginal or Torres Strait Islander	1.4 (0.6–2.9)
Born in non‐English speaking country	1.4 (1.2–1.6)
Smoked after 20 weeks' gestation	1.9 (1.1–3.2)
Nulliparous	1.1 (1.0–1.2)
Singleton pregnancy	0.1 (0.1–0.1)
Assisted Reproductive Technology utilised	1.8 (1.5–2.1)
Socio‐economic quintile	1.3 (1.1–1.6)
1 (most disadvantaged)	1.0 (0.9–1.3)
2	1.2 (1.0–1.4)
3	1.0 (0.9–1.2)
4	1.1 (1.0–1.3)
5 (least disadvantaged)	Reference
Rurality	
Major city	Reference
Inner regional	1.0 (0.8–1.2)
Outer regional	0.9 (0.7–1.2)
Rural and remote	1.0 (0.6–2.0)
Diabetes (pre‐existing or gestational)	0.5 (0.4–0.7)
Hypertension	0.3 (0.2–0.5)
Preeclampsia	1.1 (0.8–1.6)

Abbreviations: BMI, body mass index; CI, confidence interval; OR, adjusted odds ratio.

## 4 Discussion

### 4.1 Main Findings

We used population‐level linked administrative data to compare outcomes and costs between standard maternity care and private obstetric‐led care, matching for measurable demographic, socio‐economic and clinical differences. Additional neonatal adverse outcomes in standard maternity care included 778 more stillbirths or neonatal deaths, 2301 more NICU admissions and 2747 more APGAR scores < 7 at 5 min. Additional maternal adverse outcomes in standard maternity care included 3273 more women with 3rd or 4th degree tears and 10 627 haemorrhages. More induction of labour, fewer caesarean sections, fewer births between 34 and 36 weeks and fewer births between 37 and 39 weeks occurred in standard maternity care. Standard maternity care costs were almost $6000 per birth higher than private obstetric‐led care, with additional annual net costs of $1.77 billion to government if all women in private obstetric‐led care were to move to standard maternity care. An intention to treat analysis for those who started in private obstetric‐led care showed consistent results, as did bootstrapping, multiple sensitivity and sub‐group analyses. On the overall matched cohort, maternal vulnerabilities including higher BMI, mode of birth across induction and caesarean section and standard maternity care were associated with stillbirth and neonatal death.

### 4.2 Strengths and Limitations

Limitations include a lack of clarity on specific drivers of the observed differential outcomes, as current administrative data does not provide insights on the complexity of models of care, local protocols for care delivery or the experience levels of staff from trainees to consultants across providers and settings, acknowledging high variability in standard maternity care. Whilst the key strength of this study was the robust matching to account for broad measurable differences, generating two balanced groups for comparison, this did limit analysis to the matched sample only. Importantly, despite well‐balanced populations on measurable factors, the inability to identify individual socio‐economic status (an area‐based measure of socio‐economic status was available and utilised in the analysis), health literacy, level of education, perinatal mental health and other unmeasured social determinants of health, is likely to play a role in the differences and warrants further research. For example, interventions to reduce spontaneous preterm birth are known to reduce preterm birth at maximum by 20% [7], therefore, further exploration of the factors influencing the observed effect is warranted. A strength is the use of administrative data to uniformly measure actual costs for births in the models of care, including all funding sources, improving accurate capture of reported costs [2].

### 4.3 Interpretation

Our findings indicate the need for further research to identify and mitigate driving factors and to optimise models of care and outcomes with and for all women [20]. Indeed, service failures from the United Kingdom and Australia are documented when there is a failure to learn and improve, with calls for embedded, iterative real‐time research in maternity care [2, 4, 5]. The data available for the current study spanned 2016–2019, emphasising the need for timely, independent, transparent and accessible deidentified health data in a Clinical Quality Registry, allowing for adjustment, matching and advanced analytics, and shown to underpin improved models of care and outcomes [21]. However, accessible data alone are insufficient to improve healthcare. Learning Health Systems incorporating plurality of evidence from women and healthcare providers, research and guidelines, data and practice and implementation and economic research and evaluation are required for improvement [2, 22]. Ongoing maternity service failures, rising eco‐social vulnerabilities and obesity, inequities and disparities as demonstrated here, mandate transparent investigation, learning and improvement with and for women, with this work now initiated in Australia.

To our knowledge, this is the first report on value‐based differences in matched populations across outcomes and costs for private obstetric‐led care. Since 2000, research has suggested lower complication rates in private compared to public hospitals in Australia. In unmatched populations, a prior Australian study identified higher rates of major birth injury or tears in women, and higher neonatal resuscitation, NICU admission and death in public compared to private hospitals [23]. Another unmatched study reported higher rates of adverse outcomes following caesarean section in public compared to private care [24]. After adjusting for some confounders, higher rates of perinatal deaths in public compared to private hospitals were also reported [25], with another in low‐risk women showing mixed findings [26]. Other studies failed to find differences for non‐continuity obstetric care compared to midwifery care in public hospitals [27, 28, 29]. In an unmatched single public hospital study, continuity of midwifery care, private obstetric care and standard maternity care showed differences in APGARs < 7 (2.1%, 3.4% and 2.3% respectively) and neonatal death or stillbirth (0.41%, 1.73% and 1.05% respectively) [30]. Randomised trials of continuity of midwifery care models compared to other public models of care have shown lower costs and equivalent or better outcomes [6], with a Cochrane review showing lower caesarean section and induction rates and lower costs, without significant differences in foetal death rates [6]. In the current study, we advance knowledge with a large population sample drawn from multiple states and robustly matched, showing comparatively lower adverse events in private obstetric‐led care. Potential drivers may include clear provider responsibility, improved continuity of care and stronger relational aspects between women and providers [31]. The totality of this evidence mandates action to identify drivers of observed differences and to embed research and improvement in maternity care to address these and improve outcomes.

In the context of the continued rising rates, the public health system in Australia, the United Kingdom and elsewhere has focused on reducing caesarean sections [3, 32]. Here, we found that caesarean section was associated with a lower risk of stillbirth or neonatal death, compared to other modes of birth. This needs to be balanced with known other adverse outcomes [33, 34]. We also note that in private obstetric‐led care, caesarean section birth, birth between 34 and 36 weeks and birth between 37 and 39 weeks were higher; however, total costs in this setting were lower. The lower costs could be attributable to fewer adverse outcomes, reducing high‐cost care (such as NICU admission or more complex admission) in private obstetric‐led care and the different funding rates for private obstetric‐led care and standard maternity care. For example, in public hospitals caesarean section births are funded at a higher rate than vaginal births; but for private obstetricians, funding for caesarean sections and vaginal birth were set at equal amounts. In contrast, we found that induction of labour was associated with over twice the risk of stillbirth or neonatal death. In the context of rising maternal risks, these findings mandate further research on the optimal mode of birth and indications for specific interventions, supported by shared decision‐making tools for healthcare providers and women.

Our study also identified other correlates of adverse outcomes including rapidly rising maternal obesity, driven by eco‐social factors. Public health initiatives are vital here, as individual lifestyle interventions for preconception obesity have not been proven to be effective and sustainable, whilst other medical treatment options offer promise; these require further research. In pregnancy, however, concerted public health initiatives such as a sugar tax, already implemented in over 100 countries including the United Kingdom, are proven to reduce gestational weight gain and adverse pregnancy outcomes [35]. Likewise, supportive lifestyle interventions during pregnancy, embedded into maternity care, are supported by level I evidence on efficacy and cost savings, with recommendations for implementation [36].

## 5 Conclusion

Improving equity and value across costs and outcomes is a fundamental priority in maternity care. Using unique population‐level, linked datasets, we have demonstrated significantly worse outcomes for women and neonates, and higher costs, in standard maternity care compared to private obstetric‐led care, controlling for measurable demographic, socio‐economic and clinical characteristics. We have also shown that maternal factors, mode of birth and models of care are correlates of neonatal death. Overall, this study highlights the need for further research, embedded in clinical care. Given the potential disparities in outcomes between models of care, we propose that a National Maternity Learning Health System and Clinical Quality Registry are vital to embed implementation and comparative effectiveness research in routine practice, identify underlying drivers and enable rapid improvement in models of care and outcomes for all women.

## Author Contributions

E.J.C. conceived the original study idea and undertook the analysis. E.J.C. and H.T. drafted the manuscript. E.J.C., J.E., B.W.M. and H.T. co‐designed the analysis. All authors contributed to the interpretation of the findings and edited the final manuscript.

## Ethics Statement

For Maternity1000, ethics approval was obtained from the Townsville Hospital and Health Service Human Research Ethics Committee (HREC) (HREC/16/QTHS/223), James Cook University HREC (H7246) and the Australian Institute of Health and Welfare HREC (EO2017‐1‐338). We also obtained Public Health Act Approval (RD007377). The Maternity2000 project received human research ethics approval from the New South Wales Health Service Human Research Ethics Committee (HREC) (HREC/ETH00684/2020.11) and the Australian Institute of Health and Welfare Ethics Committee (EO2020/4/1167). We also received Public Health Act Approval (PHA 20‐00684). No identifiable patient information was provided to the authors.

## Conflicts of Interest

The authors declare no conflicts of interest.

## Supporting information


**Appendix S1.** Health service funding in Australia.
**Table S1.** Sensitivity analysis of neonatal and maternal birth outcomes based upon women’s characteristics, matched cohort of women giving birth in standard maternity care and private obstetric‐led care in three states, 2016–2019.
**Table S2.** Sensitivity analysis of neonatal birth outcomes based upon neonate’s characteristics, matched cohort of women giving birth in standard maternity care and private obstetric‐led care in three states, 2016–2019.
**Table S3.** Outcomes from matched cohort of women giving birth with standard maternity care and private obstetric‐led care in three states 2016–2019.
**Table S4.** Sensitivity analysis—Odds ratio of neonatal and maternal birth outcomes, whole, unmatched cohort of women giving birth in standard maternity care and private obstetric‐led care in three states, 2016–2019, adjusted for age, body mass index (BMI), born in a non‐English speaking country, socio‐economic status, rurality of residence, identification as Aboriginal or Torres Strait Islander, smoking after 20 weeks’ gestation, parity, plurality, Assisted Reproductive Technology (ART) use, and pre‐existing diabetes, gestational diabetes, hypertension or preeclampsia.
**Table S5.** Stepped logistic regression model of odds ratio of stillbirth or neonatal death, women giving birth in standard maternity care and private obstetric‐led care in three states, 2016–2019.
**Table S6.** Demographic characteristics of women before and after matching, all women giving birth in one state, 2016–2018.
**Table S7.** Sensitivity analysis of cost per birth, matched cohort of women giving birth in standard maternity care and private obstetric‐led care in one state, 2016–2018, stratified by socio‐economic status.
**Table S8.** Cost per birth to different funders, matched cohort of women giving birth in standard maternity care and private obstetric‐led care in one state, 2016–2018, stratified by socio‐economic status.
**Table S9.** Number of stillbirths and neonatal deaths that occurred in different birthweight centile groups, gestational age groups, those with congenital anomalies and those with an APGAR score of < 4 at 5 min, in the matched cohort of women giving birth in standard maternity care and private obstetric‐led care in three states, 2016–2019.

## Data Availability

Our data access approvals specifically prohibit any data sharing, and thus data sharing is not possible.
