# Supplementary material for: Maternal and Neonatal Outcomes and Health System Costs in Standard Public Maternity Care Compared to Private Obstetric‐Led Care: A Population‐Level Matched Cohort Study
Source: BJOG. 2025 Jul 14;133(1):72–82. doi: 10.1111/1471-0528.18286 (PMC12676196; doi:10.1111/1471-0528.18286)
Supplement: Supplementary file 1 — Appendix S1. Health service funding in Australia. Table S1. Sensitivity analysis of neonatal and maternal birth outcomes based upon women’s characteristics, matched cohort of women giving birth in standard maternity care and private obstetric‐led care in three states, 2016–2019. Table S2. Sensitivity analysis of neonatal birth outcomes based upon neonate’s characteristics, matched cohort of women giving birth in standard maternity care and private obstetric‐led care in three states, 2016–2019. Table S3. Outcomes from matched cohort of women giving birth with standard maternity care and private obstetric‐led care in three states 2016–2019. Table S4. Sensitivity analysis—Odds ratio of neonatal and maternal birth outcomes, whole, unmatched cohort of women giving birth in standard maternity care and private obstetric‐led care in three states, 2016–2019, adjusted for age, body mass index (BMI), born in a non‐English speaking country, socio‐economic status, rurality of residence, identification as Aboriginal or Torres Strait Islander, smoking after 20 weeks’ gestation, parity, plurality, Assisted Reproductive Technology (ART) use, and pre‐existing diabetes, gestational diabetes, hypertension or preeclampsia. Table S5. Stepped logistic regression model of odds ratio of stillbirth or neonatal death, women giving birth in standard maternity care and private obstetric‐led care in three states, 2016–2019. Table S6. Demographic characteristics of women before and after matching, all women giving birth in one state, 2016–2018. Table S7. Sensitivity analysis of cost per birth, matched cohort of women giving birth in standard maternity care and private obstetric‐led care in one state, 2016–2018, stratified by socio‐economic status. Table S8. Cost per birth to different funders, matched cohort of women giving birth in standard maternity care and private obstetric‐led care in one state, 2016–2018, stratified by socio‐economic status. Table S9. Number of stillbirths and neonatal deat [file BJO-133-72-s001.docx]

**Appendix S1: Health service funding in Australia**

All care outside of public hospitals is provided by private providers with Federal government subsidy through Medicare and usually out-of-pocket fees for patients. Pharmaceuticals are Federal government subsidised through the Pharmaceutical Benefits Scheme (PBS), with out-of-pocket fees for some patients. Care within public hospitals care is provided through hospitals that are government managed and funded jointly by the Federal and state governments through Public Hospital Funding Agreements, with no out-of-pocket fees to patients. Care within private hospitals, which are privately managed, is subsidised through Medicare plus with out-of-pocket fees to patients. Individuals may elect to take out private health insurance to cover the patient out-of-pocket fee component.

**Table S1: Sensitivity analysis of neonatal and maternal birth outcomes based upon women’s characteristics, matched cohort of women giving birth in standard maternity care and private** **obstetric led care** **in three states 2016 – 2019.**

|  | ***Low risk, Nulliparous Women*** | ***Low risk, Multiparous Women*** | ***Nulliparous Women*** | ***Multiparous Women*** | ***Nulliparous Women, >37 weeks*** | ***Multiparous Women, >37 weeks*** |
| --- | --- | --- | --- | --- | --- | --- |
| ***OUTCOMES*** | ***OR (95% CI)*** | ***OR (95% CI)*** | ***OR (95% CI)*** | ***OR (95% CI)*** | ***OR (95% CI)*** | ***OR (95% CI)*** |
| Neonatal death or stillbirth | 2.0 (1.6 – 2.5) | 2.1 (1.7 – 2.6) | 2.0 (1.8 – 2.3) | 1.9 (1.7 – 2.2) | 2.0 (1.3 – 3.1) | 2.4 (1.7 – 3.5) |
| Stillbirth | 1.7 (1.4 – 2.2) | 2.0 (1.5 – 2.5) | 1.8 (1.5 – 2.1) | 1.7 (1.5 – 2.0) | 1.9 (1.2 – 3.2) | 2.2 (1.4 – 3.4) |
| Neonatal death | 3.7 (2.3 – 6.1) | 2.7 (1.6 – 4.3) | 3.0 (2.3 – 2.8) | 2.7 (2.1 – 3.4) | 2.1 (0.8 – 5.6) | 2.9 (1.6 – 5.4) |
| Neonatal Intensive Care Admission^ | 2.9 (2.4 – 3.5) | 2.5 (2.1 – 3.0) | 2.9 (2.6 – 3.2) | 2.8 (2.6 – 3.1) | 3.8 (3.0 – 4.8) | 4.4 (3.6 – 5.3) |
| APGAR Score <7 at 5mins | 1.7 (1.6 – 1.9) | 2.3 (2.1 – 2.6) | 1.9 (1.8 – 2.0) | 2.2 (2.1 – 2.3) | 2.3 (2.0 – 2.7) | 2.7 (2.4 – 3.1) |
| APGAR Score <4 at 5mins | 1.8 (1.5 – 2.1) | 2.5 (2.1 – 2.9) | 1.9 (1.7 – 2.1) | 2.2 (2.0 – 2.5) | 2.1 (1.5 – 2.9) | 2.9 (2.3 – 3.6) |
| Birthweight, <5th centile | 1.4 (1.3 – 1.4) | 1.4 (1.2 – 1.5) | 1.4 (1.3 – 1.5) | 1.3 (1.3 – 1.4) | 1.8 (1.7 – 2.0) | 1.9 (1.8 – 2.1) |
| Birthweight, 5th-10th centile | 1.1 (1.1 – 1.2) | 1.1 (1.0 – 1.2) | 1.1 (1.1 – 1.2) | 1.1 (1.1 – 1.2) | 1.3 (1.2 – 1.4) | 1.4 (1.3 – 1.5) |
| Trauma | 4.8 (2.0 – 11.5) | 1.7 (0.7 – 4.3) | 3.5 (2.3 – 5.6) | 2.5 (1.5 – 4.1) | 4.3 (1.8 – 10.1) | 3.1 (1.2 – 7.8) |
| Hypoxia | 4.6 (3.2 – 6.8) | 6.5 (3.6 – 11.6) | 4.2 (3.3 – 5.4) | 7.3 (5.0 – 10.4) | 3.5 (1.9 – 6.6) | 7.3 (3.5 – 15.0) |
| Other | 1.9 (1.8 – 2.1) | 1.5 (1.4 – 1.7) | 1.8 (1.8 – 1.9) | 1.6 (1.5 – 1.6) | 1.9 (1.7 – 2.1) | 1.7 (1.6 – 1.9) |
| Perineal damage^#^ | 2.8 (2.6 – 3.0) | 3.3 (2.9 – 3.7) | 3.5 (3.2 – 3.7) | 4.2 (3.7 – 4.7) | 3.7 (3.2 – 4.4) | 4.9 (3.8 – 6.5) |
| Maternal haemorrhage | 2.9 (2.7 – 3.1) | 2.6 (2.4 – 2.7) | 2.9 (2.8 – 3.0) | 2.5 (2.4 – 2.6) | 2.8 (2.6 – 3.0) | 2.6 (2.5 – 2.8) |
| Retained placenta | 3.3 (3.7 – 4.0) | 2.8 (2.3 – 3.5) | 3.8 (3.3 – 4.4) | 3.4 (3.0 – 3.9) | 4.3 (3.3 – 5.5) | 4.4 (3.5 – 5.5) |

^Neonatal intensive care admission data for one state not available; # Perineum damage denominator excludes those who had a planned caesarean section; caesarean section – unplanned denominator excludes births with planned caesarean section.

**Table S2: Sensitivity analysis of neonatal birth outcomes based upon neonate’s characteristics, matched cohort of women giving birth in standard maternity care and private** **obstetric led care in three states, 2016 – 2019**

|  | ***<=28 weeks gestation*** | ***29 – 32 weeks gestation*** | ***33 – 36 weeks gestation*** | ***>=37 weeks gestation*** | ***<28 weeks gestation with congenital abnormalities*** | ***>28 weeks gestation with congenital abnormalities*** | ***<28 weeks gestation without congenital abnormalities*** | ***29 – 32 weeks gestation without congenital abnormalities*** | ***33 – 36 weeks gestation without congenital abnormalities*** | ***>=37 weeks gestation without congenital abnormalities*** |
| --- | --- | --- | --- | --- | --- | --- | --- | --- | --- | --- |
| ***OUTCOMES*** | ***OR (95% CI)*** | ***OR (95% CI)*** | ***OR (95% CI)*** | ***OR (95% CI)*** | ***OR (95% CI)*** | ***OR (95% CI)*** | ***OR (95% CI)*** | ***OR (95% CI)*** | ***OR (95% CI)*** | ***OR (95% CI)*** |
| Neonatal death or stillbirth | 2.4 (2.1 – 2.8) | 0.8 (0.6 – 1.1) | 1.9 (1.5 – 2.4) | 2.2 (1.7 – 3.0) | 1.3 (0.8 – 2.0) | 0.9 (0.6 – 1.4) | 3.2 (2.6 – 4.0) | 0.8 (0.5 – 1.3) | 1.2 (0.9 – 1.7) | 2.0 (1.3 – 3.0) |
| Stillbirth | 1.6 (1.4 – 1.8) | 0.8 (0.2 – 1.2) | 1.8 (1.4 – 2.4) | 2.1 (1.5 – 2.9) | 0.8 (0.5 – 1.1) | 1.5 (0.8 – 2.6) | 2.1 (1.7 – 2.7) | 0.9 (0.5 – 1.7) | 1.4 (0.9 – 2.0) | 2.0 (1.2 – 3.3) |
| Neonatal death | 3.0 (2.4 – 3.9) | 0.8 (0.4 – 1.5) | 2.1 (1.3 – 3.6) | 2.6 (1.6 – 4.4) | 2.5 (1.4 – 4.4) | 0.7 (0.4 – 1.1) | 3.6 (2.5 – 5.0) | 0.5 (0.2 – 1.4) | 0.8 (0.4 – 1.8) | 2.0 (1.0 – 4.3) |
| Neonatal Intensive Care Admission^ | 3.4 (2.8 – 4.2) | 2.5 (1.9 – 3.2) | 2.8 (2.5 – 3.2) | 4.2 (3.6 – 4.8) | 0.8 (0.6 – 1.3) | 1.2 (1.0 – 1.4) | 5.2 (4.1 – 6.7) | 2.7 (2.1 – 3.6) | 2.9 (2.5 – 3.2) | 4.5 (3.8 – 5.3) |
| APGAR Score <7 at 5mins | 2.9 (2.5 – 3.4) | 1.1 (0.8 – 1.4) | 1.9 (1.7 – 2.2) | 2.5 (2.3 – 2.8) | 1.4 (0.9 – 2.2) | 1.3 (1.0 – 1.7) | 4.4 (3.6 – 5.4) | 1.0 (0.7 – 1.4) | 1.6 (1.4 – 1.9) | 2.2 (1.9 – 2.5) |
| APGAR Score <4 at 5mins | 2.1 (1.8 – 2.4) | 0.9 (0.7 – 1.3) | 2.2 (1.8 – 2.7) | 2.5 (2.1 – 3.1) | (0.8 – 1.8) | 1.2 (0.8 – 1.9) | 3.0 (2.4 – 3.7) | 0.9 (0.5 – 1.6) | 1.6 (1.2 – 2.2) | 2.1 (1.5 – 2.9) |
| Birthweight, <5th centile | 1.0 (0.7 – 1.3) | 1.4 (0.8 – 2.6) | 1.7 (1.5 – 2.0) | 1.9 (1.8 – 2.0) | Not reportable | 1.0 (0.8 – 1.2) | 0.9 (0.5 – 1.7) | 0.6 (0.3 – 1.3) | 1.6 (1.3 – 2.0) | 1.6 (1.5 – 1.8) |
| Birthweight, 5th-10th centile | (0.4 – 0.8) | 1.3 (0.7 – 2.3) | 1.3 (1.1 – 1.5) | 1.4 (1.3 – 1.5) | Not reportable | 1.1 (0.9 – 1.4) | 0.4 (0.2 – 0.8) | 0.8 (0.4 – 1.7) | 1.2 (1.0 – 1.4) | 1.3 (1.2 – 1.4) |
| Trauma | Not reportable | Not reportable | 2.0 (0.6 – 6.8) | 3.8 (2.0 – 7.1) | Not reportable | Not reportable | Not reportable | Not reportable | Not reportable | 4.8 (2.0 – 11.2) |
| Hypoxia | Not reportable | Not reportable | 1.1 (0.5 – 2.2) | 5.0 (3.1 – 8.0) | Not reportable | Not reportable | Not reportable | Not reportable | Not reportable | 4.6 (2.6 – 7.9) |
| Other | Not reportable | Not reportable | 1.6 (1.5 – 1.7) | 1.8 (1.7 – 1.9) | Not reportable | Not reportable | Not reportable | Not reportable | Not reportable | 1.6 (1.5 – 1.8) |

**Table S3: Outcomes from matched cohort of women giving birth with standard maternity care and private obstetric led care in three states 2016 *–* 2019**

| ***OUTCOMES*** | **Standard maternity care (n=188,493)** | | **Private obstetric led care (n=188,493)** | | ***OR (95% CI)*** |
| --- | --- | --- | --- | --- | --- |
|  | ***N*** | ***%*** | ***N*** | ***%*** |  |
| ***Socioeconomic Quintile 1 (Most disadvantaged)*** | | | | | |
| Neonatal death or stillbirth | 151 | 1.9% | 68 | 0.5% | 2.3 (1.7 – 3.1) |
| Neonatal Intensive Care Admission^ | 161 | 2.6% | 105 | 1.7% | 1.4 (1.1 – 1.8) |
| APGAR Score <7 at 5mins | 455 | 3.3% | 223 | 1.6% | 2.1 (1.8 – 2.5) |
| Hypoxia | 42 | 0.3% | 21 | 0.2% | 2.9 (1.5 – 5.6) |
| Other | 775 | 5.5% | 515 | 3.7% | 1.5 (1.4 – 1.7) |
| Perineal damage^#^ | 332 | 2.4% | 105 | 0.8% | 2.6 (2.0 – 3.2) |
| Maternal haemorrhage | 1285 | 9.2% | 569 | 4.1% | 2.5 (2.3 – 3.8) |
| Retained placenta | 90 | 0.6% | 42 | 0.2% | 2.4 (1.6 – 3.5) |
| ***Socioeconomic Quintile 2*** | | | | | |
| Neonatal death or stillbirth | 182 | 0.8% | 99 | 0.5% | 1.9 (1.5 – 2.5) |
| Neonatal Intensive Care Admission^ | 255 | 2.3% | 157 | 0.9% | 2.6 (2.0 – 3.3) |
| APGAR Score <7 at 5mins | 665 | 3.1% | 366 | 1.7% | 1.9 (1.7 – 2.2) |
| Hypoxia | 93 | 0.4% | 22 | 0.1% | 4.4 (2.4 – 8.0) |
| Other | 1138 | 5.2% | 830 | 3.8% | 1.5 (1.4 – 1.6) |
| Perineal damage^#^ | 542 | 2.5% | 210 | 1.0% | 2.5 (2.1 – 3.0) |
| Maternal haemorrhage | 2204 | 10.2% | 1006 | 4.6% | 2.4 (2.2 – 2.6) |
| Retained placenta | 219 | 1.0% | 89 | 0.4% | 3.2 (2.4 – 4.2) |
| ***Socioeconomic Quintile 3*** | | | | | |
| Neonatal death or stillbirth | 377 | 1.0% | 179 | 0.5% | 2.2 (1.8 – 2.6) |
| Neonatal Intensive Care Admission^ | 587 | 2.7% | 247 | 1.2% | 2.5 (2.2 – 3.0) |
| APGAR Score <7 at 5mins | 1240 | 3.2% | 547 | 1.4% | 2.3 (2.1 – 2.6) |
| Hypoxia | 123 | 0.3% | 35 | 0.1% | 3.2 (2.1 – 4.7) |
| Other | 2262 | 5.8% | 1418 | 3.7% | 1.6 (1.5 – 1.7) |
| Perineal damage^#^ | 1081 | 2.8% | 342 | 0.9% | 2.5 (2.2 – 2.8) |
| Maternal haemorrhage | 3584 | 9.3% | 1505 | 3.9% | 2.6 (2.4 – 2.8) |
| Retained placenta | 244 | 0.6% | 140 | 0.4% | 1.8 (1.4 – 2.2) |
| ***Socioeconomic Quintile 4*** | | | | | |
| Neonatal death or stillbirth | 382 | 0.8% | 210 | 0.4% | 1.9 (1.6 – 2.2) |
| Neonatal Intensive Care Admission^ | 1151 | 3.7% | 412 | 1.3% | 3.1 (2.8 – 3.5) |
| APGAR Score <7 at 5mins | 1344 | 2.8% | 701 | 1.4% | 1.9 (1.8 – 2.1) |
| Hypoxia | 170 | 0.4% | 32 | 0.1% | 9.0 (5.5 – 14.9) |
| Other | 2929 | 6.0% | 1882 | 3.9% | 1.6 (1.5 – 1.7) |
| Perineal damage^#^ | 1260 | 2.6% | 316 | 0.7% | 3.3 (2.9 – 3.7) |
| Maternal haemorrhage | 4624 | 9.5% | 2021 | 4.1% | 2.5 (2.4 – 2.6) |
| Retained placenta | 618 | 1.3% | 209 | 0.4% | 3.3 (2.8 – 3.9) |
| ***Socioeconomic Quintile 5 (Least disadvantaged)*** | | | | | |
| Neonatal death or stillbirth | 496 | 0.8% | 267 | 0.4% | 1.8 (1.6 – 2.1) |
| Neonatal Intensive Care Admission^ | 1408 | 4.0% | 548 | 1.6% | 3.0 (2.7 – 3.3) |
| APGAR Score <7 at 5mins | 1813 | 2.8% | 938 | 1.5% | 2.0 (1.8 – 2.1) |
| Hypoxia | 219 | 0.3% | 48 | 0.1% | 6.0 (4.2 – 8.6) |
| Other | 3642 | 5.7% | 2010 | 3.1% | 1.9 (1.8 – 2.0) |
| Perineal damage^#^ | 1453 | 2.3% | 384 | 0.6% | 3.1 (2.8 – 3.5) |
| Maternal haemorrhage | 6045 | 9.4% | 2136 | 3.3% | 3.2 (3.0 – 3.3) |
| Retained placenta | 1088 | 1.7% | 210 | 0.3% | 5.6 (4.8 – 4.2) |

**Table S4: Sensitivity analysis - Odds Ratio of neonatal and maternal birth outcomes, whole, unmatched cohort of women giving birth in standard maternity care and private** **obstetric led care** **in three states 2016 – 2019, adjusted for age, body mass index (BMI), born in a non-English speaking country, socio-economic status, rurality of residence, identification as Aboriginal or Torres Strait Islander, smoking after 20 weeks’ gestation, parity, plurality, Assisted Reproductive Technology (ART) use, and pre-existing diabetes, gestational diabetes, hypertension or preeclampsia**

|  | **Standard maternity care compared to private obstetric led care** |
| --- | --- |
| ***OUTCOMES*** | ***OR (95% CI)*** |
| Neonatal death or stillbirth | 1.6 (1.5 – 1.8) |
| Stillbirth | 1.4 (1.3 – 1.6) |
| Neonatal death | 2.2 (1.8 – 2.6) |
| Neonatal Intensive Care Admission | 3.0 (2.8 – 3.2) |
| APGAR Score <7 at 5mins | 1.7 (1.6 – 1.8) |
| APGAR Score <4 at 5mins | 1.7 (1.6 – 1.9) |
| Birthweight, <5th centile | 1.3 (1.2 – 1.3) |
| Birthweight, 5th-10th centile | 1.1 (1.0 – 1.1) |
| Trauma | 3.7 (2.6 – 5.4) |
| Hypoxia | 5.4 (4.4 – 6.8) |
| Other | 1.6 (1.6 – 1.7) |
| Perineal damage | 3.3 (3.0 – 3.5) |
| Perineal damage – 3^rd^ degree tear | 3.3 (3.1 – 3.6) |
| Perineal damage – 4^th^ degree tear | 2.6 (2.0 – 3.5) |
| Maternal haemorrhage | 2.8 (2.7 – 2.8) |
| Ruptured uterus | 2.6 (1.7 – 3.9) |
| Retained placenta | 4.5 (4.1 – 4.9) |

**Table S5: Stepped logistic regression model of odds ratio of stillbirth or neonatal death, women giving birth in standard maternity care and private** **obstetric led care** **in three states 2016 – 2019**

|  | ***OR (95% CI)*** | ***OR (95% CI)*** | ***OR (95% CI)*** |
| --- | --- | --- | --- |
| Standard maternity care, compared to private obstetric led care | 2.0 (1.8 – 2.1) | 2.1 (1.9 – 2.3) | 1.7 (1.5 – 1.8) |
| Preterm birth, (<37 weeks gestation) |  | 72.4 (64.8 – 81.0) | 98.7 (88.1 – 110.5) |
| Caesarean section birth |  |  | 0.2 (0.1 – 0.2) |

**Table S6: Demographic characteristics of women before and after matching, all women giving birth in one state, 2016- 2018**

|  | **Pre-Matching** | | | | **Post-Matching** | | | |
| --- | --- | --- | --- | --- | --- | --- | --- | --- |
|  | **Standard maternity care (n= 110,041)** | | **Private obstetric led care (n=38,425)** | | **Standard maternity care (n= 33,857)** | | **Private obstetric led care (n=33,857)** | |
|  | **N** | **%** | **N** | **%** | **N** | **%** | **N** | **%** |
| Age group |  |  |  |  |  |  |  |  |
| 20 and under | 3295 | 3.0% | 35 | 0.1% | 30 | 0.1% | 30 | 0.1% |
| 21-34 years | 85124 | 77.4% | 24837 | 64.6% | 23277 | 68.2% | 23277 | 68.2% |
| 35 years and over | 21622 | 19.7% | 13553 | 35.3% | 10825 | 31.7% | 10825 | 31.7% |
| Mean age (SD) | 29.5 (5.7) | | 33.0 (4.4) | | 31.0 (5.5) | | 32.7 (4.2) | |
| BMI Group | | | | | | | | |
| Underweight | 8100 | 7.4% | 1782 | 4.6% | 1569 | 4.6% | 1569 | 4.6% |
| Healthy range | 52562 | 47.8% | 22373 | 58.2% | 19987 | 58.6% | 19987 | 58.6% |
| Overweight | 25185 | 22.9% | 8686 | 22.6% | 7661 | 22.5% | 7661 | 22.5% |
| Obese | 24194 | 22.0% | 5584 | 14.5% | 4915 | 14.4% | 4915 | 14.4% |
| Mean BMI (SD) | 26.0 (6.5) | | 24.8 (5.2) | | 24.8 (5.3) | | 25.1 (5.7) | |
| Born in non-English speaking country | 6852 | 19.5% | 6852 | 17.8% | 6326 | 18.5% | 6326 | 18.5% |
| Aboriginal or Torres Strait Islander | 8576 | 7.8% | 226 | 0.6% | 174 | 0.5% | 174 | 0.5% |
| Socioeconomic quintile | | | | | | | | |
| 1 (Most disadvantaged) | 10720 | 9.7% | 734 | 1.9% | 2332 | 6.9% | 2332 | 6.9% |
| 2 | 13470 | 12.2% | 1739 | 4.5% | 3946 | 11.7% | 3946 | 11.7% |
| 3 | 10048 | 9.1% | 2129 | 5.5% | 8519 | 25.2% | 8519 | 25.2% |
| 4 | 9336 | 8.5% | 2151 | 5.6% | 10461 | 30.9% | 10461 | 30.9% |
| 5 (least disadvantaged) | 11365 | 10.3% | 3800 | 9.9% | 8584 | 25.4% | 8584 | 25.4% |
| Rurality | | | | | | | | |
| Major city | 68541 | 62.3% | 28024 | 72.9% | 24719 | 72.4% | 24719 | 72.4% |
| Inner regional area | 21235 | 19.3% | 5570 | 14.5% | 5031 | 14.7% | 5031 | 14.7% |
| Outer regional area | 16986 | 15 4% | 4195 | 10.9% | 3867 | 11.3% | 3867 | 11.3% |
| Rural and remote | 3200 | 2.9% | 602 | 1.6% | 495 | 1.5% | 495 | 1.5% |
| Smoked after 20 Weeks | 12699 | 11.6% | 189 | 0.5% | 156 | 0.5% | 156 | 0.5% |
| Nulliparous | 31805 | 28.9% | 13655 | 35.5% | 11642 | 34.1% | 11642 | 34.1% |
| Singleton Pregnancy | 107112 | 97 3% | 37060 | 96.5% | 33344 | 97.7% | 33344 | 97.7% |
| Assisted Reproductive Technology utilised | 3253 | 3.0% | 5168 | 13.5% | 2303 | 6.8% | 2303 | 6.8% |
| Diabetes (Pre-existing or gestational) | 15002 | 13.6% | 3398 | 8.8% | 3024 | 8.9% | 3024 | 8.9% |
| Hypertension | 2968 | 2.7% | 1213 | 3.2% | 935 | 2.7% | 935 | 2.7% |
| Preeclampsia | 2681 | 2.4% | 871 | 2.3% | 624 | 1.8% | 624 | 1.8% |

BMI= Body Mass Index

**Table S7: Sensitivity analysis of cost per birth, matched cohort of women giving birth in standard** **maternity care and private obstetric led care in one state, 2016 – 2018, stratified by socioeconomic status**

| **Costs** | **Standard maternity care** | **Private obstetric led care** |
| --- | --- | --- |
|  | Mean (95% CI) | Mean (95% CI) |
| *Low risk, Nulliparous* | | |
| Total cost | $26,863 (26,267 – 27,464) | $21,506 (21,211 – 21,802) |
| *Low risk, Multiparous* | | |
| Total cost | $25,986 (25,559 – 26,413) | $20,944 (20,744 – 21,145) |
| *Excluding births with a NICU admission* | | |
| Total cost | $27,306 (27,148 – 27,464) | $22,492 ((22,387 – 22,596) |
| *Based on model of care at 28 weeks, one state, only births >28 weeks* |  |  |
| Total cost | $28,391 (20,104 – 31,448) | $23,432 (18,628 – 25,479) |

**Table S8: Cost per birth to different funders, matched cohort of women giving birth in standard maternity care and private obstetric led care in one state, 2016 – 2018, stratified by socioeconomic status**

| ***Socioeconomic Quintile*** | **Standard maternity care (n= 33,857)** | **Private obstetric led care (n=33,857)** |
| --- | --- | --- |
|  | **Mean (95% CI)** | **Mean (95% CI)** |
| Quintile 1 (Most disadvantaged) | $29,437 (28,314 – 30,559) | $22,525 (22,035 – 23,015) |
| Quintile 2 | $29,300 (28,635 – 29,966) | $22,465 (22,154 – 22,775) |
| Quintile 3 | $29,583 (29,072 – 30,095) | $22,759 (22,528 – 22,990) |
| Quintile 4 | $28,280 (27,886 – 28,674) | $23,176 (22,862 – 23,490) |
| Quintile 5 (Least disadvantaged) | $27,659 (27,288 – 28,030) | $22,452 (22,260 – 22,643) |

**Table S9: Number of stillbirths and neonatal deaths that occurred in different birthweight centile groups, gestational age groups, those with congenital anomalies, and those with an APGAR score of <4 at 5 minutes, in the matched cohort of women giving birth in standard maternity care and private obstetric led care in three states, 2016 – 2019**

|  | **Stillbirth and Neonatal Death** | | **Stillbirth** | | **Neonatal Death** | |
| --- | --- | --- | --- | --- | --- | --- |
|  | *Public* | *Private* | *Public* | *Private* | *Public* | *Private* |
| *Gestational age birthweight centile* | | | | | | |
| <=5th centile | 200 | 73 | 146 | 59 | 54 | 14 |
| 6th-10th centile | 62 | 41 | 49 | 35 | 13 | 6 |
| 11th-25th centile | 204 | 93 | 163 | 80 | 41 | 13 |
| >25^th^ centile | 1118 | 599 | 745 | 460 | 373 | 139 |
| *Gestational age at birth* |  |  |  |  |  |  |
| <=28 weeks | 1030 | 494 | 696 | 397 | 334 | 97 |
| 29 - 32 weeks | 126 | 58 | 99 | 45 | 27 | 13 |
| 33 - 36 weeks | 197 | 119 | 156 | 97 | 41 | 22 |
| >=37 weeks | 231 | 135 | 152 | 95 | 79 | 40 |
| *Congenital Abnormalities* |  |  |  |  |  |  |
| Yes | 567 | 364 | 385 | 268 | 182 | 96 |
| No | 274 | 162 | 181 | 120 | 93 | 42 |
| *APGAR <4, 5 mins* |  |  |  |  |  |  |
| Yes | 115 | 71 | Not applicable | | 114 | 71 |
| No | 726 | 455 |  |  | 161 | 67 |
